# Retinal Vessel Density in Optical Coherence Tomography Angiography in Optic Atrophy after Nonarteritic Anterior Ischemic Optic Neuropathy

**DOI:** 10.1155/2017/9632647

**Published:** 2017-02-19

**Authors:** Chun-Hsiu Liu, Ling-Yuh Kao, Ming-Hui Sun, Wei-Chi Wu, Henry Shen-Lih Chen

**Affiliations:** Department of Ophthalmology, Chang Gung Memorial Hospital, Chang Gung University College of Medicine, Taoyuan, Taiwan

## Abstract

*Aims*. To compare optical coherence tomography angiography (OCT-A) retinal vasculature measurements between normal and optic atrophy after nonarteritic anterior ischemic optic neuropathy (NAION) subjects. *Design*. This prospective observational study was conducted between July 2015 and August 2016 at the ophthalmology outpatient department of a referral center in Taiwan. Peripapillary (4.5 × 4.5 mm) and parafoveal (6 × 6 mm) OCT-A scans were acquired. Measurements of the peripapillary region were obtained in two areas: (1) circumpapillary vessel density (cpVD) and (2) whole enface image vessel density (wiVD). *Results*. 13 participants with optic atrophy after NAION had lower peripapillary vessel density than the 18 age-matched participants in the healthy control (HC) group (*p* < 0.001 for both cpVD and wiVD). However, the parafoveal vessel density was not significantly different between the two groups (*p* = 0.49). The areas under the receiver operating characteristic curve for the HC and NAION eyes were 0.992 for cpVD and 0.970 for wiVD. cpVD and wiVD were significantly correlated with the average retinal nerve fiber layer thickness (*p* < 0.001 for both). *Conclusion*. Peripapillary retinal perfusion is significantly decreased in optic atrophy after NAION. OCT-A may aid in the understanding of structure-function-perfusion relationships in NAION.

## 1. Introduction

Nonarteritic anterior ischemic optic neuropathy (NAION) is the most common and visually threatening optic nerve disorder in the middle-aged and elderly population. Though the exact pathophysiology of NAION is unclear, it is generally thought to result from infarction of retrolaminar portion of the optic nerve head which is supplied by the short posterior ciliary arteries [1].

Radial peripapillary capillaries (RPCs) are a superficial layer of capillaries located in the peripapillary region of the retina that extend outward from the optic disc [2]. The vessels follow the course of the nerve fibers and supply the retinal ganglion cell axons. Some functional and structural changes, including arcuate scotoma and flame-shaped retinal hemorrhage matching the distribution of this network, imply the possible relationship between RPCs and certain optic neuropathies such as glaucoma [3, 4]. Fluorescein angiography (FA) is currently the standard way to identify the impaired circulation and its location in NAION. However, the RPC change after NAION has rarely been analyzed in clinical practice because of its ineffective visualization with FA due to the presence of choroidal fluorescence.

Optical coherence tomography (OCT) is a noninvasive imaging technique that is commonly used in the clinical diagnosis of many ocular diseases. Recently, Jia et al. developed a new method using high-speed OCT to measure local circulation [5]. Using the split-spectrum amplitude-decorrelation angiography (SSADA) algorithm, blood flow and the microvascular network can be visualized by decorrelating the motion of red blood cells from the static tissues without the need for contrast injection. Spaide et al. proved that OCT angiography (OCT-A) with SSADA can be used to image the RPCs and the inner and outer retinal plexuses effectively [6]. A detailed search of the published literature did not reveal any studies concerning quantitative measurement of OCT-A in NAION patients. To the best of our knowledge, this is the first study using OCT-A in the investigation of NAION patients. The purpose of this study was to measure the retinal perfusion in optic atrophy after NAION with OCT-A and to determine the relationship of SSADA-based RPCs density with retinal nerve fiber layer (RNFL) thickness and visual field (VF) changes in NAION.

## 2. Materials and Methods

### 2.1. Study Population

This prospective observational study was conducted between July 2015 and August 2016. Patients with NAION and the healthy control (HC) group were recruited from our ophthalmology clinic at Chang Gung Memorial Hospital (Taoyuan, Taiwan). The research protocols were approved by the institutional review board and adhered to the tenets of the Declaration of Helsinki.

All of the patients were interviewed regarding their medical history and underwent a comprehensive ophthalmic evaluation, including best-corrected visual acuity (BCVA) and refraction assessments, slit-lamp biomicroscopy, intraocular pressure (IOP) measurement, optic nerve head (ONH) evaluation and fundus examination, digital color fundus photography (Digital Non-Mydriatic Retinal Camera, Canon, Tokyo, Japan), automated visual field assessment (Humphrey 30-2 SITA standard strategy, Carl Zeiss Meditec, Jena, Germany), spectral-domain OCT (SD-OCT), and OCT-A. Systemic blood pressure was measured by sphygmomanometry in an upright sitting position after a 10 min rest period.

The inclusion criteria for the NAION group included all of the following: (1) history of a sudden onset of painless vision loss; (2) a VF defect consistent with NAION; (3) optic disc edema at onset that was confirmed by at least one neuro-ophthalmology expert; (4) no evidence of other ocular diseases such as glaucoma, arteritic anterior ischemic optic neuropathy, optic neuritis, or coexisting retinal pathologies that may cause the visual symptoms; and (5) resolution of the initial optic disc and retinal edema. If both eyes of a patient satisfied these criteria, the eye with longer elapsed time between the onset of disease and measurements was selected for analysis to minimize the bias associated with optic disc swelling.

HC eyes were defined as follows: (1) BCVA better than 20/20; (2) IOP less than 21 mmHg; (3) normal-appearing ONH and RNFL; (4) symmetric ONH between the right and left eyes; (5) no evidence of retinal pathology or optic neuropathy; (6) normal Humphrey SITA 30-2 VF; and (7) no history of intraocular surgery. Only one eye per person was randomly selected for analysis.

The exclusion criteria for all eyes were as follows: (1) age less than 20 years or more than 80 years; (2) refractive error greater than +3.0 diopter or less than −6.0 diopter; (3) previous intraocular surgery; (4) any disease other than NAION that may cause a VF defect or optic disc abnormalities; (5) inability to perform reliably on automated VF testing or poor cooperation in OCT imaging studies; and (6) poor image quality with a signal strength index less than 45 and registered image sets with residual motion artifacts.

### 2.2. OCT Angiography Data Acquisition and Processing

OCT-A using the SD-OCT system (RTVue-XR Avanti; Optovue, CA, USA) enables simultaneous assessment of the ocular structure and microvasculature. Details regarding the use of this system have been described by Jia et al. [5] and Liu et al. [7]. The RNFL thickness was averaged from a 3.4 mm diameter circular sampling profile centered on the disc. The ganglion cell complex (GCC) thickness was averaged over a 7 mm diameter circular area centered on the fovea consisting of the inner retinal layer composed of the macular nerve fiber layer, ganglion cell layer, and inner plexiform layer.

**Figure 1:**
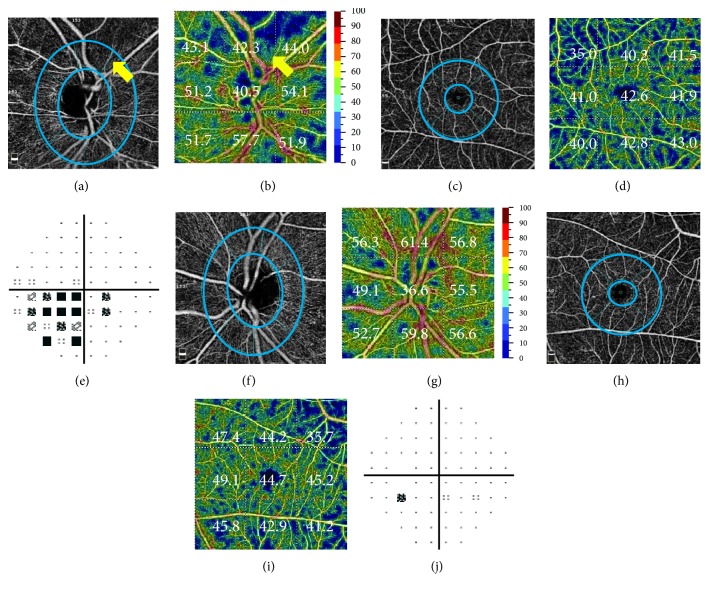
Comparison of the peripapillary and parafoveal retinal angiograms: (a), (f) en face optical coherence tomography angiograms (OCT-A) in the radial peripapillary capillary (RPC) layer, (b), (g) vessel density (%) of PRCs with a color-coded map, (c), (h) OCT-A in the parafoveal area, (d), (i) vessel density (%) in the parafoveal area with a color-coded map, and (e), (j) total deviation of the visual field in a representative nonarteritic ischemic optic neuropathy (NAION) eye and the normal fellow eye. In the NAION eye (a)–(e), the microvascular network of the RPC layer was attenuated (arrow) compared with that of the normal fellow eye (f)–(j). The parafoveal microvascular networks in these examples showed no clear differences. The circles indicate the measured area for circumpapillary and parafoveal vessel density.

The OCT-A images were acquired using an 840 nm superluminescent diode and a bandwidth of 45 nm. Optic disc (4.5 × 4.5 mm) and macular (6 × 6 mm) OCT-A scans were acquired by two repeated B-scans, each of which consisted of 304 A-scans. To quantify the vessel densities in the peripapillary retina, the optic disc boundary was determined based on the scanning laser ophthalmoscopy images. The vessel density was estimated by the percentage area occupied by the large vessels and microvasculature in the target regions and was measured using the installed flow density mapping software AngioAnalytics. We measured vessel density in two retinal regions: the peripapillary and parafoveal areas (Figure 1). The measurements of the peripapillary region were obtained in two areas as follows: whole enface image vessel density (wiVD) was calculated in the entire 4.5 × 4.5 mm image, and circumpapillary vessel density (cpVD) was estimated in the region defined as a 750 *μ*m wide elliptical annulus extending from the optic disc boundary. The parafoveal vessel density (pfVD) was measured in the region defined as an annulus with an outer diameter of 3 mm and an inner diameter of 1 mm centered at the fovea.

The quality of all OCT-A images was assessed. Poor-quality images with a signal strength index less than 45, registered image sets with residual motion artifacts visible as discontinuous irregular vessel patterns or disc boundaries, and images with a local weak signal on the en face angiogram were excluded from the analysis.

### 2.3. Statistical Analysis

All statistical analyses were performed using SPSS software version 19.0 (SPSS, Inc., Chicago, Illinois, USA). The distribution of numerical data was tested for normality by the Shapiro-Wilk test. The baseline characteristics and differences in the clinical features between the NAION group and HC group were compared for statistical significance using the Mann-Whitney test for continuous data and chi-square test for categorical data. To determine the relationship between vessel density, RNFL and GCC thicknesses, and VF change, the Pearson correlation coefficient and multivariate regression analysis were calculated. The diagnostic accuracy for differentiating between NAION and HC eyes was evaluated by calculating the area under the receiver operating characteristic (AUROC) curve. A *p* value less than 0.05 was considered statistically significant.

## 3. Results

Thirteen eyes from 13 Taiwanese subjects with optic atrophy after NAION and 18 eyes from 18 age-matched HC subjects were included in the analysis. The mean interval between the onset of disease and measurement was 14.1 months. Table 1 summarizes the demographics and clinical characteristics. There was no significant difference in age, IOP, and refraction between the two groups. The systolic and diastolic blood pressures and prevalence of hypertension were significantly higher in the NAION group. The mean deviation (MD) of VF was −16.4 dB in the NAION group. The mean RNFL and GCC thicknesses were significantly thinner in the NAION group than those in the HC group (79.3 *μ*m versus 98.3 *μ*m and 75.3 *μ*m versus 102.5 *μ*m, resp., *p* < 0.05 for both).

The OCT-A scan provides en face reflectance images and measurements of peripapillary and parafoveal perfusion. Example images of NAION and normal eyes are shown in Figure 1. The peripapillary microvascular network assessed by en face angiography and color-mapped imaging showed attenuated vessel density in the NAION eyes and a denser microvascular network in the normal fellow eye, although the parafoveal microvascular network showed no obvious difference. The total deviation maps of VF showed inferonasal depression matching the location of the perfusion defect in the NAION eyes.

**Figure 2:**
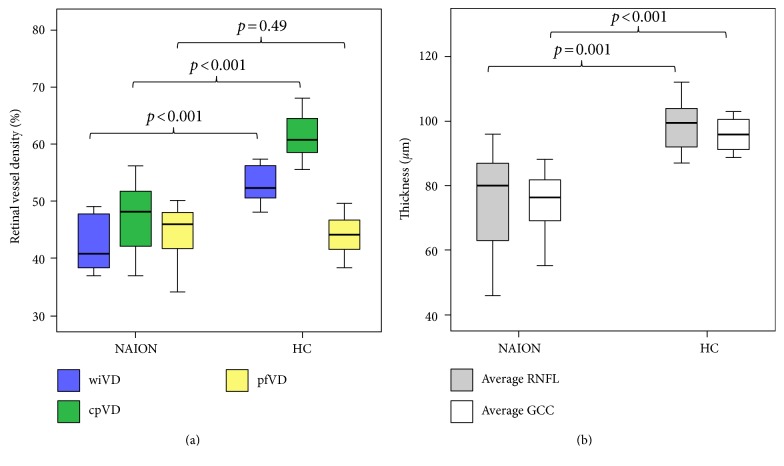
Box plots showing the retinal vessel density and thickness in nonarteritic ischemic optic neuropathy (NAION) eyes and healthy control (HC) eyes: (a) whole enface image vessel density (wiVD), circumpapillary vessel density (cpVD), and parafoveal vessel density (pfVD); (b) average retinal nerve fiber layer (RNFL) thickness and average ganglion cell complex (GCC) thickness. The median (dark bold line), IQR (box), and the whole range of values (whiskers) are shown. Mann-Whitney tests showed significant reduction in the wiVD, cpVD, average RNFL thickness, and average GCC thickness of the NAION group compared to the HC group.

The mean wiVD and cpVD in NAION eyes were 43.9% and 48.3%, respectively, which were significantly lower than those in the HC group (*p* < 0.001 for both) (Table 2 and Figure 2). However, the pfVD was not significantly different between the two groups (44.9% versus 44.1%, *p* = 0.49). The AUROC for discriminating NAION from normal eyes was highest for the average GCC thickness, followed by the cpVD, wiVD, and average RNFL thickness (1.000, 0.992, 0.970, and 0.909, resp.).

Table 3 shows the Pearson correlations of the vessel density of the peripapillary area, average RNFL and GCC thicknesses, and VF function in NAION eyes. Both cpVD and wiVD had significant correlation with MD, average RNFL thickness, and average GCC thickness (*p* < 0.05 for all). In the multiple linear regression analysis where wiVD and cpVD were considered the dependent variables, only the average RNFL thickness was a predictor of wiVD and cpVD (*p* = 0.013 and 0.018, resp.). Other factors including age, gender, blood pressure, refraction, IOP, MD, and average GCC were not significant explanatory variables in the multivariate models.

## 4. Discussion

In this study, we found decreased peripapillary retinal perfusion in optic atrophy after NAION, a finding that is correlated with RNFL thinning. This is the first study using OCT-A to demonstrate the peripapillary retinal perfusion changes in optic atrophy after NAION.

OCT-A is a newly developed technique and has been used to visualize retinal vascular changes, mainly in retinal diseases. The peripapillary area of the retinal blood flow is a relatively unexplored field. Using OCT-A, peripapillary retinal perfusion has been shown to be decreased in glaucoma and correlated with VF damage [7–9]. Decreased retinal perfusion in the optic nerve head has also been reported in patients with multiple sclerosis [10]. In NAION eyes, our results are compatible with those of previous studies using different devices. Blood flow in the ONH and retina has been reported to be decreased in optic atrophy using laser Doppler techniques [11]. Wang et al., using Doppler OCT, also found decreased peripapillary retinal blood flow in NAION eyes [12]. Although the exact cause is unknown, there are several possible reasons for the decreased peripapillary retinal perfusion in NAION eyes. First, the neurodegenerative changes following an episode of NAION cause a reduction of the number of nerve fibers in the ONH and therefore decrease metabolic demand and blood flow via autoregulatory mechanisms [10]. This explanation is supported by the significant correlation between the peripapillary retinal perfusion and the thickness of RNFL in the present study. Second, the decreased peripapillary retinal perfusion could be a primary result of an ischemic event in NAION. RPCs possess a unique role in supporting the nerve fiber layer around the disc [2]. Compared with other retinal capillaries, RPCs are longer and straighter, and they connect large-caliber arteries to the large venous trunk directly, without anastomosis. These anatomical peculiarities make RPCs more sensitive to decreased blood flow than other retinal capillaries. According to Hayreh, the pathogenic concept of NAION is acute hypoperfusion of the optic nerve head [13]. It is reasonable that this acute hypotension may also cause damage to the RPC directly. However, this hypothesis requires further studies to prove.

There is no significant difference in parafoveal retinal perfusion between the NAION and HC groups in the present study. Because previous studies have reported that thinning of the macula and ganglion cell layer occurs as early as one month following the onset of NAION [14, 15], this result was not expected. One possible reason for this result is due to the different composition of central and peripheral retina. The decreased peripapillary retinal perfusion is thought to be secondary to the atrophy of RNFL which is more prominent in the peripapillary area. On the contrary, the neurosensorial retinal layer thickness in the fovea centralis is reported to be similar to control values in optic atrophy after NAION [16], and thus, the parafoveal retinal perfusion is less affected.

In the present study, the AUROC of the peripapillary vessel density is comparable to that of traditional structural analyses by OCT. It is worth mentioning that we found the diagnostic ability of cpVD to be similar to that of wiVD. This result was unexpected because wiVD was previously reported to have better diagnostic accuracy than cpVD for differentiating between healthy and glaucoma groups [8]. One possible explanation for this result is, because that study recruited early glaucoma patients, vessel dropout associated with focal RNFL damage may occur in the retina farther from the circumpapillary region [17] and can be better covered by larger measurement area. On the contrary, the distribution of thinning of RNFL in the eyes after NAION is more widespread, and thus, cpVD and wiVD demonstrate similar diagnostic ability.

The optic disc blood flow in NAION was previously investigated using various methods. Using a laser Doppler flowmeter, Leiba et al. demonstrated that the ONH blood flow in NAION eyes is significantly lower than that in healthy control eyes [18]. However, a laser Doppler flowmeter is not sufficiently sensitive to measure the detailed changes of small vessels. FA is currently the preferred method for imaging the retinal circulation. Hayreh has described the correlation of the segmental disc with adjacent choroidal filling delay during the early stage [13]. However, FA is considered an invasive procedure and does not provide objective quantitative measurements.

OCT-A offers several advantages over other imaging techniques. OCT-A visualizes the vasculature using red blood cells as the intrinsic contrast; thus, it not only eliminates the risk of allergic reaction due to dye injection but is also less affected by intrinsic tissue reflectance. OCT-A can provide quantitative measurement of vascularity at different layers. Compared with other methods, the high repeatability and reproducibility of OCT-A make it more suitable for monitoring the change in vascularity in the ONH.

This study has several limitations. The sample size was relatively small. However, we used G^∗^power (Faul et al., freely available online) [19] to calculate the prospective power and to determine the required minimal sample size based on another similar study using OCT-A in glaucoma [7] before conducting the present study. It supported that the sample size in this study is sufficient to test the hypothesis at significance level 0.05 and power 0.8. There is marked interindividual variation in the blood supply of the ONH; differences may exist between individuals and ethnic groups that restrict the external validity of the current study. In addition, we did not include patients with acute-stage disease to prevent the bias associated with optic disc swelling. However, because the objective of OCT-A is to measure the dynamic blood flow within the vessel instead of the vessel itself, it may be less affected by optic disc edema. Further studies with more subjects in the acute stage of disease may help elucidate the role of RPCs in NAION.

In conclusion, our study demonstrated significantly decreased peripapillary retinal perfusion in optic atrophy after NAION that is correlated with thinning of RNFL thickness. OCT-A may help to elucidate the structure-function-perfusion relationships in NAION.

## Figures and Tables

**Table 1:** Demographic and clinical data of NAION patients and controls^b^.

	NAION (*n* = 13)	Control (*n* = 18)	*p* value
Demographic characteristics			
Age, year	59.0 (10.7)	51.4 (14.1)	0.19
Male, number (%)	6 (46)	7 (39)	1.00
Ocular characteristics			
IOP, mmHg	13.3 (1.7)	13.5 (2.9)	0.73
SE, diopters	0.7 (2.6)	−1.1 (4.2)	0.35
MD, dB	−16.4 (7.4)	−0.2 (4.2)	0.03^a^
Clinical characteristics			
Systolic BP, mmHg	135.5 (16.7)	116.2 (11.7)	0.03^a^
Diastolic BP, mmHg	82.6 (13.3)	56.6 (28.1)	0.03^a^
Self-reported history of diabetes, *n* (%)	4 (31)	1 (6)	0.14
Self-reported history of hypertension, *n* (%)	6 (46)	1 (6)	0.03^a^

NAION: nonarteritic anterior ischemic optic neuropathy; IOP: intraocular pressure; SE: spherical equivalent; MD: mean deviation; BP: blood pressure.

^a^Statistically significant.

^b^Unless otherwise indicated, data are given as the mean (standard deviation).

**Table 2:** Mean values and diagnostic accuracy (AUROC) of vessel density and RNFL and GCC thicknesses^b^.

Diagnostic parameters	NAION eyes (*n* = 13)	Controls (*n* = 18)	*p* value	95% CI of the difference	AUROC
wiVD, %	43.9 (6.4) [37.1~58.9]	53.6 (3.4) [48.2~59.5]	<0.001^a^	−13.8~−5.6	0.970
cpVD, %	48.3 (7.7) [37.0~64.9]	61.6 (3.0) [55.6~68.1]	<0.001^a^	−18.1~−8.4	0.992
pfVD, %	44.9 (4.9) [34.2~50.2]	44.1 (3.5) [38.5~49.7]	0.49	−3.0~4.4	0.409
Average RNFL thickness, *μ*m	79.3 (27.8) [46.0~152.0]	98.3 (7.6) [87.0~112.0]	0.001^a^	−35.5~−2.6	0.909
Average GCC thickness, *μ*m	75.3 (10.3) [55.2~88.1]	102.5 (29.7) [88.8~219.9]	<0.001^a^	−44.8~−9.6	1.000

NAION: nonarteritic anterior ischemic optic neuropathy; CI: confidence interval; AUROC: area under the receiver operating characteristic; wiVD: whole en face image vessel density; cpVD: circumpapillary vessel density; pfVD: parafoveal vessel density; RNFL: retinal nerve fiber layer; GCC: ganglion cell complex.

^a^Statistically significant.

^b^Unless otherwise indicated, data are given as the mean (standard deviation) [range].

**Table 3:** Pearson's correlation coefficient matrix for peripapillary vessel density, structural variables, and visual field.

		wiVD	cpVD	MD	RNFL thickness	GCC thickness
wiVD	*r*	1				
	*p*					
cpVD	*r*	0.950	1			
	*p*	<0.001^a^				
MD	*r*	0.502	0.624	1		
	*p*	0.05^a^	0.01^a^			
Average RNFL thickness	*r*	0.777	0.784	0.266	1	
	*p*	<0.001^a^	<0.001^a^	0.32		
Average GCC thickness	*r*	0.425	0.499	0.598	0.454	1
	*p*	0.02^a^	0.004^a^	0.01^a^	0.01^a^	

wiVD: whole en face image vessel density; cpVD: circumpapillary vessel density; MD: mean deviation; RNFL: retinal nerve fiber layer; GCC: ganglion cell complex.

^a^Statistically significant.
